# Complete genome sequence of *Streptomyces* sp. strain VNUA24, a potential antifungal bacterium isolated from soil

**DOI:** 10.1128/MRA.00372-23

**Published:** 2023-08-29

**Authors:** Tam Thi Thanh Dang, Son Truong Dinh, Thu Thi Nguyen, Hai Thanh Nguyen, Canh Xuan Nguyen

**Affiliations:** 1 Faculty of Biotechnology, Vietnam National University of Agriculture, Hanoi, Vietnam; University of Maryland School of Medicine, Baltimore, Maryland, USA

**Keywords:** *Streptomyces *sp*.*, genome sequencing

## Abstract

*Streptomyces* species are important resources for secondary metabolites, including antimicrobial compounds. The *Streptomyces* sp. strain VNUA24 was isolated from a soil sample in Vietnam. The strain was identified as having antifungal activity against numerous fungal pathogens. Whole-genome sequencing was done to explore the biocontrol capability of this strain.

## ANNOUNCEMENT

*Streptomyces* are renowned for their extraordinary capacity to create useful secondary metabolites (1). To investigate the capacity of *Streptomyces* sp. strain VNUA24 as a biocontrol agent, the complete genome of this species was sequenced, assembled, and annotated. The wild-type strain VNUA24 of *Streptomyces* sp. was isolated from a soil sample collected at the banana field (18°36*'*15.7*"* N, 105°49*'*05.4*"* E) in Ha Tinh province, Vietnam.

A single colony of the isolated strain was grown on 30 mL of Gause’s No. 1 medium at 30°C with shaking at 200 rpm for 2 days for DNA extraction. Genomic DNA (gDNA) was extracted from 15 mL of culture using a modified DNA isolation procedure (2). The concentration, integrity, and purity of gDNA were determined by fluorometer and 1% agarose gel electrophoresis. Then, the gDNA sample was sent to the Beijing Genomics Institute (BGI, Shenzhen, China) for sequencing with the DNBSEQ and PacBio Sequel II platforms. On the DNBSEQ platform, a qualified single-strand circle DNA library was constructed and sequenced by BGISEQ-500 following the company’s protocol (3). Finally, 150 bp pair-end reads were generated. Next, the raw reads with a certain portion of low-quality, ambiguous bases, adapter contamination, or duplication contamination were removed using SOAPnuke v1.5.5 software (https://github.com/BGI-flexlab/SOAPnuke) (4). On the PacBio platform, genomic DNA was sheared (about 10–15 Kb) with Covaris g-TUBEs. The library of *Streptomyces* sp. strain VNUA24 was prepared using the SMRT bell template prep kit v1.0 (Pacific Bioscience) according to the manufacturer’s instructions. The PacBio subread set was produced using four single-molecule real-time (SMRT) cells with *Zero-mode waveguide* sequencing arrays. The PacBio subreads with a length of less than 1 kb were discarded. The program Canu v1.5 was used for self-correction (https://github.com/marbl/canu/releases) (5). Draft genomic unitigs were assembled using Canu, a high-quality corrected circular consensus sequence subreads set. To improve the accuracy of the genomic sequences, GATK v1.6-13 (https://www.broadinstitute.org/gatk/) was used for single-base corrections (6). The genome was trimmed and circularized using Circlator v1.5.5 (7). Glimmer 3.02 software (http://www.cbcb.umd.edu/software/glimmer/) with Hidden Markov models was used to predict genes (8, 9). For tRNA, rRNA, and sRNA recognition, the RNAmmer v1.2, tRNAscan-SE v1.3.1 (10), and the Rfam v9.1 database (11) were utilized. Taxonomy was predicted using the Type (Strain) Genome Server (TYGS) platform (12). Default parameters were used for all analyses in this research unless otherwise noted.

The DNBSEQ sequencing obtained 8,764,210 paired-end sequence reads and 131× coverage. The Pacbio sequencing yielded 3,453,834,249 reads, 346× coverage, an *N*_50_ value of 9,090 bp, and the longest read of 209,286 bp. The *Streptomyces* sp. strain VNUA24 genome contains a single circular chromosome (9,974,491 bp) with a G + C content of 71.83% and 9,020 predicted genes. Genome annotation revealed a total of 67 tRNAs and 18 rRNAs. Taxonomic classification according to TYGS databases revealed that strain VNUA24 could be a new species of the genus *Streptomyces*.

## Data Availability

The whole-genome sequence and associated data have been deposited at GenBank (accession number CP119143) and the Sequence Read Archive (SRR23728162 and SRR23728163).
